# Efficient Anaerobic Digestion of Microalgae Biomass: Proteins as a Key Macromolecule

**DOI:** 10.3390/molecules23051098

**Published:** 2018-05-06

**Authors:** Jose Antonio Magdalena, Mercedes Ballesteros, Cristina González-Fernandez

**Affiliations:** 1 Biotechnological Processes Unit, IMDEA Energy, 28040 Madrid, Spain; joseantonio.magdalena@imdea.org (J.A.M.); mercedes.ballesteros@imdea.org (M.B.); 2 Biofuels Unit, CIEMAT, 28040 Madrid, Spain

**Keywords:** microalgae, anaerobic digestion, proteins, biogas, inhibition

## Abstract

Biogas generation is the least complex technology to transform microalgae biomass into bioenergy. Since hydrolysis has been pointed out as the rate limiting stage of anaerobic digestion, the main challenge for an efficient biogas production is the optimization of cell wall disruption/hydrolysis. Among all tested pretreatments, enzymatic treatments were demonstrated not only very effective in disruption/hydrolysis but they also revealed the impact of microalgae macromolecular composition in the anaerobic process. Although carbohydrates have been traditionally recognized as the polymers responsible for the low microalgae digestibility, protease addition resulted in the highest organic matter solubilization and the highest methane production. However, protein solubilization during the pretreatment can result in anaerobic digestion inhibition due to the release of large amounts of ammonium nitrogen. The possible solutions to overcome these negative effects include the reduction of protein biomass levels by culturing the microalgae in low nitrogen media and the use of ammonia tolerant anaerobic inocula. Overall, this review is intended to evidence the relevance of microalgae proteins in different stages of anaerobic digestion, namely hydrolysis and methanogenesis.

## 1. Introduction

Environmental issues and energy self-sufficiency concerns related to fossil fuels have led to research on new approaches to improve renewable energy production to substitute them. Anaerobic digestion is one of those technologies devoted to the production of biofuels, which involves the degradation of organic matter through the action of different microorganisms. Anaerobic digestion exhibits many advantages such as its efficiency for organic matter removal, its applicability at any scale and the wide variety of substrates that can be used as feedstock. Likewise, the multiproduct generation attained during digestion is also a major benefit of this technology. Those end-products, including biogas and digestate, are easy to separate and can be a source of energy and fertilizers, respectively [1]. 

Among the different substrates that can be employed, microalgae are being recently studied since this biomass can be grown in residual effluents, do not need arable land to be cultivated while contributing to CO_2_ mitigation and wastewater bioremediation [2]. Previous studies have demonstrated the technoeconomic and environmental benefits of microalgae biomass for bioenergy purposes when considered as by-product in other technologies [3,4,5,6,7,8]. In the same manner, out of the bioenergy producing technologies where microalgae can be used as feedstocks, anaerobic digestion is probably the most economically feasible since it does not require highly concentrated biomass [9] and anaerobes can use proteins, carbohydrates and lipids for methane production purposes [10]. Microalgae biomass has a wide range of compositions, depending on growth conditions and species [11,12]. In general terms, biochemical profile of chlorophytes range 30–60% of proteins, 20–40% of carbohydrates, and 4–57% of lipids [13,14]. Each macromolecule has different achievable methane yields [10]. Thus, in principle, different microalgae compositions produce different methane yields [12]. At the same time, microalgae composition varies depending not only among strains but also on the growth conditions (nutrients availability and operational conditions) [15,16]. In addition to the different macromolecular composition that microalgae might exhibit, this biomass also differs in structural features. Most of the microalgae able to thrive in wastewater effluents have a chemically complex and structurally robust cell wall composed of low biodegradable substances that hinder the anaerobic digestion [17,18]. Some of these compounds are sporopollenin, algaenan, and polymeric carbohydrates that offer a barrier towards anaerobes [19,20]. During anaerobic digestion, cell walls are degraded by extracellular enzymes of hydrolytic bacteria. Nevertheless, this process might be too slow and thus, a limited hydrolysis rate renders the anaerobic digestion as a lengthy and inefficient bioprocess. Pretreatments are used in order to facilitate the accessibility of these extracellular enzymes whereby improving hydrolysis stage. Different microalgae pretreatments have been studied such as thermal, chemical, mechanical or biological. Methane yields improvements achieved with those different pretreatments can be found elsewhere [21,22,23,24]. Out of the different pretreatments, biological approach is the most environmentally friendly [25]. Opposite to other pretreatments, the additional benefits of biological pretreatments are the absence of inhibiting by-products [26] and the high selectivity of the reactions [27]. This approach might not only be used for biomass hydrolysis but also to provide crucial information related to the macromolecule that reduces the anaerobic biodegradability of microalgae biomass. In this manner, this review summarizes the main results attained during the last years of research devoted to microalgae pretreatments in the biogas production context. Moreover, this period of research highlighted the importance of proteins on different stages of the digestion. This review attempts to provide comprehensive evidences of the key role of microalgae proteins. 

## 2. Pretreatment of Microalgae Biomass to Improve Biogas Production

Since low biodegradability is a common issue in anaerobic digestion of different substrates (such as activated sludge, lignocellulose and photosynthetic microorganisms), a wide range of pretreatments are available to enhance the hydrolysis step [28]. Cell wall rupture or hydrolysis is needed to make available microalgae organic matter to anaerobic microorganisms [29]. Pretreatments are classified in four main groups, namely thermal, mechanical (ultrasound and microwave), chemical (acidic, alkaline, solvents and ozonation) and thermo-chemical (acid or alkali reagents addition combined with high temperatures) and biological (enzymes and microorganisms). Those pretreatments have been intensively studied during the last decade to improve biogas production of microalgae biomass (Table 1). Most of them have been assessed in Biochemical Methane Potential (BMP) assays (batch digestion mode).

### 2.1. High Energy Demanding Pretreatments

Thermal, thermo-chemical and mechanical pretreatments are considered as high energy demanding processes and, in order to evaluate its efficiency, the final energy balance of the pretreatment process has to be addressed. Given that thermal energy is available in biogas production facilities, the most used pretreatment is thermal application. Thermal pretreatments involve biomass heat up in a wide range of temperatures (50–270 °C) and reaction time (from minutes to hours). With regard to thermal application, the effect on the biomass depends on the microalgae strain and applied temperature [30]. Passos et al. [31] and Passos and Ferrer [42] applied thermal pretreatment to *Scenedesmus* sp. biomass at 75 °C and 95 °C for 10 h resulting in methane yield enhancement of 58% and 69%, respectively . Similar values were attained by González-Fernández et al. [43] when treating *Scenedesmus* at 80 °C for only 15 min, highlighting the impact of temperature rather than the heating time as the most relevant parameter in thermal pretreatment. Similar temperatures were tested in *Chlorella* biomass (70 and 90 °C) for 30 min resulting in an enhanced methane yield of 37% and 48% compared to the raw biomass (322 mL CH_4_/g VS_in_) [32]. These results evidenced that thermal pretreatments are strain specific and thus, at the same temperature applied, different methane yields enhancement can be attained among the different biomass used. Higher temperatures (130 °C for 15–30 min) were also tested, resulting in 28% methane yield increase when compared to a raw biomass composed by a mixture of green algae (*Stigeoclonium* sp. and *Monoraphidium* sp.) and diatoms (*Nitzschia*) (105 mL CH_4_/g VS_in_) [31]. Due to the potential formation of Maillard compounds at higher temperatures, moderate temperatures in the range of 80–120 °C are most widely tested. Moreover, thermal pretreatments have been tested not only in batch mode, but also in semicontinuous mode. Méndez et al. [33] reported a methane yield enhancement of 1.5-fold compared to raw *Chlorella* biomass (84 mL CH_4_/g COD_in_) when using 120 °C for 40 min for feeding a Completely Stirred Tank Reactor (CSTR). Although no common inhibitors were identified, the results obtained in the CSTR were considerably lower (50% less) than the ones obtained in batch mode digestion. This experimentation corroborated the need to test each pretreatment in different feeding modes. Although thermal pretreatments normally present positive results in terms of methane yield, the values attained are very diverse depending on different variables such as the pretreated biomass, temperature, pretreatment time employed and operation mode during the digestion. Moreover, as mentioned above, these methods involved some drawbacks such as the formation of recalcitrant compounds that could potentially decrease the performance of the process [34,35]. 

Mechanical pretreatments are commonly employed to disrupt different kind of organic substrates in industrial processes [44,45]. Ultrasound treatment has been applied to disrupt microalgae cell wall in different bioprocess devoted to biofuel production, such as ethanol production from *Chlorella* biomass [46] and biodiesel generation from *Spirulina* biomass [47]. In the case of anaerobic digestion, ultrasound pretreatment has also shown positive results in terms of methane yield enhancement. González-Fernández et al. [43] applied 128.9 kJ/g TS at 85 °C and 30 min to enhance methane yield of *Scenedesmus* biomass from 81 mL CH_4_/g COD_in_ to 153 mL CH_4_/g COD_in_ (87% enhancement). Nevertheless, those authors also pointed out the fact that ultrasound application is having associated an increase in temperature which also acts as a pretreatment. As a matter of fact, when it comes to the pretreatment of *Scenedesmus* sp., the benefits of ultrasound application were rather questionable compared to the enhancement in methane yield attained only with the application of temperature. Ultrasound pretreatment (26.7 KJ/g TS for 30 min) was also applied to *Monoraphidium* sp. and *Stigeoclonium* sp. biomass and their methane yields were enhanced from 105 mL CH_4_/g COD_in_ to 196 mL CH_4_/g COD_in_ [42]. When testing different energy inputs (10; 27; 40; 57 KJ/g TS), applied to different mixtures of microalgae biomass (mixture A: 40% *Chlamydomonas*, 20% *Scenedesmus* and 40% *Nannocloropsis*; mixture B: 58% *Acutodesmus obliquus*, 36% *Oocystis* sp., 1% *Phormidium* and 5% *Nitzschia* sp; Mixture C: *Microspora* ≈ 100%), an increase in methane yield ranging from 6 to 24% at 10 MJ/kg TS was determined, while higher energy inputs did not report any significant increase [34]. Despite all those positive results in terms of methane yields enhancement, the main limitation of ultrasound pretreatment is the high energy input required when compared to thermal, chemical or biological methods [21]. 

Chemical methods are often combined with heat pretreatment. Thermochemical pretreatments have been less employed than thermal and mechanical pretreatments due to its potential toxicity for the anaerobes. Cell wall disruption with alkali and acid pretreatments has been tested with positive results for the production of ethanol, butanol and biomethane when using microalgae biomass as a feedstock [48,49]. Studies related to microalgae biomass solubilization using thermo-alkaline methods include for instance the use of reagents such as NaOH or CaO. Different doses of CaO (4 and 10% *w*/*w*) and different temperatures (25, 55 and 72 °C) resulted in maximum proteins and carbohydrates solubilization of 32.4% and 31.4%, respectively, and methane yield enhancement of 25% compared to the raw biomass (260 mL CH_4_/g VS_in_) at the highest temperature and lime dose tested (72 °C and 10% *w*/*w*) [50]. When using NaOH (0.5, 2 and 5% *v*/*v*) in *Chlorella* and *Scenedesmus* biomass, the conducted experiments revealed that despite of the biomass solubilisation, the methane yield enhancement was really low (10%, [36]). Thermo-acid pretreatments have been less employed than thermo-alkali. For instance, *Chlorella* biomass was heated at 120 °C either for 20 min and 40 min. Sulphuric acid addition combined with 120 °C for 40 min enhanced carbohydrates solubilization by 7-fold, although no solubilization of the protein fraction was reported. In terms of methane production, this thermo-acid pretreatment improved the methane yield from the untreated biomass from 139 mL CH_4_ g/COD_in_ to 230 mL CH_4_ g/COD_in_ [51]. Since anaerobic digestion is taking place at around pH 7, one of the main limitations of chemical pretreatments is the need to readjust the pH previously to the digestion. In this manner, chemical costs limit the use of these pretreatments. Moreover, some of the chemicals need to be removed previously to the anaerobic digestion as they can be toxic for anaerobes [27].

In conclusion, high energy demanding pretreatment methods report high values in terms of methane yield. However, they are energetically unbalanced. This means that the energy required to carry out the pretreatment is higher than the one obtained in form of biogas. This is why research has been directed towards the use of low energy demanding pretreatments

### 2.2. Low Energy Demanding Pretreatments

Compared to other pretreatments, the biological approach presents some advantages such as lower energy demand and high specificity [37]. These pretreatments include the use of suitable enzymes or microorganisms to hydrolyze microalgae biomass. Information about the cell wall composition is scarce, but necessary in order to select the most suitable enzyme for the pretreatment. For that reason, a wide range of biocatalysts have been tested. In principle, given the similarities between higher plants and microalgae, the most studied catalysts are carbohydrases. Among them, cellulases, hemicellulases, amylases and pectinases are the most tested ones [37,52]. Some other enzymatic cocktails employed for microalgae biomass hydrolysis include lysozyme (catalyzing the hydrolysis of 1,4-beta-linkages between *N*-acetylmuramic acid and *N*-acetyl-d-glucosamine residues in peptidoglycan [53]), proteases (hydrolyzing peptide bonds [39]) and laccases [25]. Overall, the best results have been evidenced by adding commercial proteases cocktails. For instance, carbohydrases and proteases were compared hydrolyzing *Chlamydomonas reinhardtii* and *Chlorella vulgaris* [38]. Enzyme doses applied for carbohydrases and proteases were 0.3 mL/g DW and 0.2 mL/g DW, respectively. The enzymatic pretreatment lasted for 5 h and results obtained after carbohydrases addition were 86% and 96% carbohydrate solubilization for *C. vulgaris* and *C. reindhartii* while in the case of protease addition both biomass resulted in 96% protein solubilization. However, the authors pointed out that despite of the high carbohydrate solubilization, only a 14% enhancement methane yield was observed in *Chlorella* biomass, whereas no improvement was observed in *Chlamydomonas*. In the case of protease pretreated biomass, methane yield was enhanced by 51% in the *C. vulgaris* and 7% for *C. reindhartii*. The reason for the low methane yield enhancement recorded for *C. reindhartii* was due to the inherent high anaerobic biodegradability of this strain (75%, 263 mL CH_4_ g/COD_in_). Methane yield is limited by the inherent methane yield that the biomass can attain. However, the kinetics might be enhanced by the use of pretreatments. More specifically, methane yield might be enhanced by protease pretreatment in the range of 1.07 to 6.3 fold depending on the targeted microalgae biomass within 10–15 days of digestion [38,40].

An alternative to improve economically the enzymatic pretreatment and avoid the addition of high cost cocktails is the addition of hydrolytic secretomes released by other microorganisms. For instance, 0.7 g/L of cellulase-secreting bacteria was added to *Chlorella vulgaris* for 48 h resulting in an increase of 18% organic matter solubilization and 2-fold methane yield compared to the raw biomass [54]. Non-specific extracellular enzymes of *Anthracophyllum discolor* were employed to disrupt the cell wall of *Botryococcus braunii*, resulting in an improvement of 60% methane yield, when enzymatic concentration of 1000 U/mL was applied [55]. Likewise, cellulolytic marine bacteria were applied to *Botryococcus braunii* and *Nannochloropsis gaditana* biomass 1:1 ratio DW resulting in a methane enhancement of 140% and 150%, respectively compared to the raw biomass [56].

As it is observed in Table 1, almost all tested pretreatments improved methane production yields although a direct linkage between solubilization and methane enhancement still requires in-depth research in continuous systems to determine the energy balance and costs of the overall process [57]. Even though this pretreatment is economically unfeasible yet, enzymatic pretreatments, targeting at specific molecules, could provide important information in order to identify which is the microalgae macromolecule hampering biogas production when using this biomass [23].

## 3. Biological Approach to Enhance Biogas Production: Enzymatic Pretreatment

Opposite to other pretreatments, biological reactions show high selectivity and absence of inhibitory compounds. Biocatalysts do not only disrupt the cell wall, but they also hydrolyze the macromolecules during biological pretreatment. As it was indicated above, these methods are energetically competitive since they require soft temperatures and smooth shaking. Different parameters must be taken into account such as pH, temperature, enzyme dose, and exposure time [21]. Given the different macromolecular composition, structural features and cell wall composition among microalgae strains, a wide range of biocatalysts can be used. Despite of the high economic cost of the enzymatic cocktails, the use of biocatalysts can provide crucial information to identify the macromolecule hampering anaerobic digestion of microalgae biomass. Moreover, the costs could be reduced either by in situ enzymes production [54,58] or by particular enzymes secreted by bacteria and fungi via sludge bioaugmentation [23,59,60].

### 3.1. Carbohydrases

Carbohydrases are in charge of hydrolysing carbohydrates polymers present within the cell wall and inside the cells into simple sugars. Since it is believed that carbohydrates are the responsible of cell wall toughness, cellulaseshave been tested in microalgae biomass to enhance the hydrolysis. Cellulases from *Trichoderma reseei* were mixed with metal oxides to treat *Chlorella* biomass resulting in glucose yield of 91% of the theoretical maximum [61]. Furthermore, enzymatic cocktails aimed at degrading the compartmentalized cell material such as amylases and amyloglucosidases have been tested to promote the efficiency of the hydrolysis step. As a matter of fact, a combination of amylases and cellulases was tested to degrade the cell wall and the cell material with acid hydrolysis in *Chlorella sorokiniana, Nannochloropsis gaditana,* and *Scenedesmus.* This treatment produced a sugar release of 128 mg/g DW, 129 mg/g DW and 60 mg/g DW, respectively against control values for the different biomass (70 mg/g DW, 20 mg/g DW and 25 mg/g DW) [62]. Carbohydrases have also been tested to facilitate lipid extraction by using exoglucanase, endoglucanase, xylanase and laccase produced by different biomass-degrading bacteria, improving lipid extraction up to 40% [63]. All those studies are mainly focused on carbohydrates solubilisation while, only recently, the biomass subjected to carbohydrases has been investigated for biogas production purposes. Ometto et al. [9] tested different enzymatic cocktails on three different biomass, namely *Scenedesmus obliquus, Chlorella sorokiniana* and *Arthrospira maxima* [5]. Out of the tested enzymatic cocktails, mixtures of cellulase plus pectinase and esterase plus protease were the most effective catalysts for organic matter hydrolysis of all three biomass. In the same manner, commercial cocktails hydrolyzing the carbohydrate fraction such as Viscozyme, Celluclast and Pectinase (from Novozymes, Bagsværd, Denmark) have been employed in *C. vulgaris* and *Scenedesmus*. The use of Viscozyme provided carbohydrate fraction solubilization of 84% and 36% for *C. vulgaris* and *Scenedesmus* respectively, while the methane yield enhancement was 1.2-fold for both of them, despite of the different biomass composition and strain [41]. This experimentation suggested that the carbohydrate fraction cannot be understood as a whole to elucidate the relation between solubilization efficiency and the methane yield achievable. Instead of this, an in-depth research must be done concerning the carbohydrates composition of microalgae cell wall. 

### 3.2. Lipases

When compared to other macromolecular constituents, lipids could be very useful substrates for anaerobic digestion due to its high potential methane yield. More specifically, theoretical methane yield for lipids is 1.014 L CH_4_/g VS compared to 0.496 and 0.415 L CH_4_/g VS for proteins and carbohydrates, respectively [10]. However, instability of the system can easily occur due to the formation of long chain fatty acids when lipids are hydrolyzed [64]. As a matter of fact, studies are mainly focused on the optimal concentration of lipids that makes possible to carry out anaerobic digestion without inhibition. In this way, it has been highlighted that lipid fraction should not be over 30% to avoid process inhibition [65]. To overcome such an inhibition, different strategies have been developed. For instance, Palatsi et al. [66] tested different recovery strategies to reduce the negative effect of long chain fatty acids by using different feeding patterns and adsorbents addition. Despite of the high lipid potential to enhance methane yield, microalgae biomass grown in wastewater does not present high lipid content [67,68]. At this point, it should be stressed that microalgae grown in residual effluents is the only feasible way to produce biofuel using this feedstock. In this manner, really limited information on lipases treatment of microalgae biomass for biogas production can be found in literature. For instance, an enzymatic mixture containing protease, α-amylase, xylanase, lipase and cellulase employed for *Rhizoclonium* biomass (filamentous green algae) hydrolysis resulted in 40% yield enhancement [69]. In this case, the mixture of enzymes made difficult the identification of the enzymatic activity responsible for such an enhancement. Ometto et al. [9] also tested esterases in different lipid rich microalgae biomass. Moreover, this investigation reported the use of esterases alone and the mixture of esterases and proteases. No biogas production was attempted for the biomass pretreated with esterases alone and thus, no conclusion could be withdrawn. Nevertheless, their work revealed that this later enzymatic mixture supported much higher organic matter solubilization than the values attained for esterases application alone, highlighting the importance of microalgae proteins. 

### 3.3. Proteases

Microalgae biomass is normally prevailing in protein content. As a matter of fact, this polymer might represent approximately 40–60% of the microalgae dry weight [24,70]. Protein fraction might be degraded by proteases since they hydrolyze peptides into amino acids. The use of proteases is receiving particular interest in last years, especially in combination with other pretreatments or other commercial enzymatic cocktails [71,72]. Some examples on the use of proteases in different microalgae biomass were evaluated in terms of organic matter solubilization and methane yields [38,39,40]. In the context of anaerobic digestion, methane yields of *C. vulgaris* and *Scenedesmus* sp. were enhanced by 2.6-fold and 1.53-fold, respectively, when pretreated with protease [39]. It is important to note that those results were attained with proteins rich biomass. More specifically, *Chlorella vulgaris* exhibited 64% protein and 22% carbohydrate content. When dealing with carbohydrate rich *C. vulgaris* biomass (39.6%), protease hydrolysis efficiency (54%) displayed higher organic matter values than carbohydrolase hydrolysis (approx. 26%). The different effect of both enzymatic cocktails was also observed in the methane yields attained by both pretreated biomass. In that case, methane yield achieved with the biomass pretreated with proteases was 137 mL CH_4_ g/COD_in_ while 65 mL CH_4_ g/COD_in_ was obtained for the biomass pretreated with carbohydrases [40]. This fact showed that even working with carbohydrate rich *C. vulgaris*, the proteolytic cocktail supported high organic matter hydrolysis and methane yields. 

Comparison of different studies regarding enzymatic pretreatments suggested that proteins are the molecules that hindered the access of anaerobic bacteria to microalgae organic matter in the anaerobic digestion process to a greater extent than carbohydrates or lipids. Therefore, the protein fraction has been carefully analyzed during the anaerobic digestion process of microalgae biomass in the subsequent section 

## 4. Biomass Proteins in Anaerobic Digestion of Microalgae

**Figure 1:**
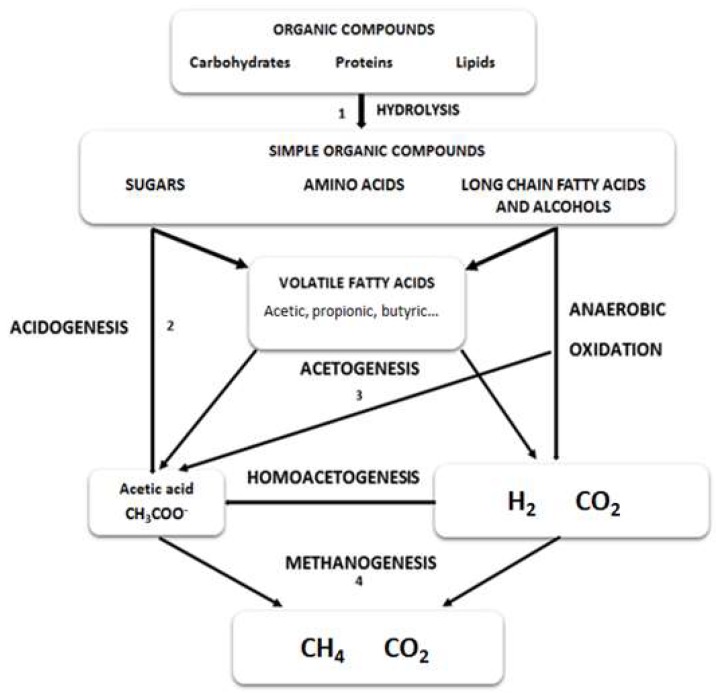
Reactive scheme for the anaerobic digestion of polymeric microalgal biomass.

Anaerobic digestion is divided in four different stages including hydrolysis, acidogenesis, acetogenesis and methanogenesis (Figure 1). When protein rich microalgae are subjected to anaerobic digestion, the bioprocess can be affected at different stages.

**Figure 2:**
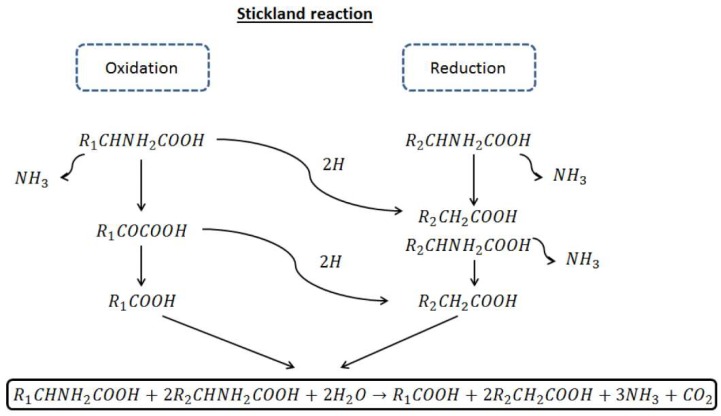
Stickland reactions scheme.

Anaerobic degradation of proteins and lipids has not been investigated in depth compared to that of carbohydrates. Proteins are hydrolyzed to aminoacids by extracellular enzymes. Anaerobic and facultatively anaerobic bacteria, mainly *Clostridium*, are responsible of aminoacids fermentation. Clostridia obtain energy by coupled oxidation-reduction reaction between aminoacids via the so-called Stickland reaction. This reaction entails the oxidation (dehydrogetation) of one aminoacid and the reduction of a second aminoacids (hydrogenation) (Figure 2). 

Aminoacids can act as electron acceptors or donors. In the first case, the aminoacid form a carboxylic acid with one carbon shorter than the original acid (e.g alanine to acetate) while when acting and electron acceptor, it retains the carbon to form a carboxylic acid with the same chain length as the original aminoacid (e.g., glycine to acetate). The aminoacid is de-ammonified by anaerobic oxidation, yielding volatile fatty acids and hydrogen, as shown in Table 2 [73].

### 4.1. The Relevance of Microalgae Proteins in the Hydrolysis Stage of Anaerobic Digestion

The first biological process involved in anaerobic digestion is hydrolysis, which is the limiting step and its effectiveness is crucial for the overall process [9,74]. Focusing on proteins, they are hydrolyzed into amino acids by extracellular enzymes secreted by different bacteria such as *Clostridium*, *Vibrio*, *Peptococcus*, *Bacillus*, *Proteus*, or *Bacteroides* [23]. As reviewed above, research devoted to microalgae digestion conducted over last years showed higher methane production in protease pretreated biomass compared to raw biomass and biomass treated with carbohydrases [40]. Methane production of protease pretreated *C. vulgaris* was enhanced by 51% compared to the raw biomass, showing the benefits of having proteins in the soluble phase. Similarly, methane yield enhancement (37%) of cyanobacteria was also attributed to the proteolytic activity developed upon biomass storage [74]. Even though protease addition has revealed the importance of microalgae proteins in microalgae digestion, it is clear that the use of commercial cocktails would not make biogas production profitable. In this manner, the use of commercial proteases helped in the identification of the macromolecule opposing more resistance to an optimal anaerobic digestion but cheaper alternatives should be investigated for avoiding the addition of commercial enzymes. Two main strategies can be applied for such a purpose. The first one entails the use of in-situ released enzymes by fungi or bacteria. Through the so-called bioaugmentation, microorganisms can be added to the anaerobic sludge used as degradation consortium. In this manner, once identified the microorganisms producing the enzymatic cocktail required for the targeted microalgae biomass, it can be added to the anaerobic sludge. Obviously, the appropriate microbial species should be carefully selected to be effective, not only for microalgae hydrolysis, but also to be viable and present good activity within the anaerobic microbiome. The potential of bioaugmentation, including the main benefits and limitations, has been recently reviewed [75]. This approach has been applied in more conventional substrates while literature available on bioaugmentation strategies devoted to microalgae anaerobic digestion is scarce. Nevertheless, this strategy was successfully applied to improve methane production of *C. vulgaris* biomass [60]. Those researchers showed an enhanced methane yield (18–38%) after adding *Clostridium thermocellum* at various inoculum ratios to degrade the carbohydrate fraction of microalgae biomass. Likewise, the same bacteria, *C. thermocellum*, was reported to enhance methane yield (18–38%) when degrading *Haematococcus pluvialis*. Therefore, this acidogenic phase bacteria is nowadays considered as a promising biotechnological tool to improve anaerobic digestion of microalgae through bioaugmentation.

The second alternative to increase the hydrolytic activity of anaerobic sludge is the use of metals. The addition of trace metals as micronutrients have been proven to stimulate methane production. The dosing needs to be well balanced to support the desired microbial activity or growth rate above which the trace metals become inhibitory or toxic. These metals are essential in the anaerobic reactions, since most of them are part of the active site of enzymes. The effect on different trace metal on anaerobic digestion can be found elsewhere [76]. Even though the use of trace elements is beneficial in most cases, the response of the system is uncertain due to the complexity of the anaerobic digestion process. It is recommended for substrates which initially have low trace element content. For instance, Kim et al. [77] evaluated the effect of trace elements at different range temperatures highlighting the benefits of using Fe, Co. or Ni for the hydrolysis step due to the increase of COD solubilization and organic acids production. 

### 4.2. The Relevance of Microalgae Proteins in the Methanogenesis Stage of Anaerobic Digestion 

Out of the subsequent stages involved in anaerobic digestion, hydrogen and acetic acid are converted to methane gas and carbon dioxide during methanogenesis. This last stage is performed by archaea. When compared to anaerobic bacteria involved in anaerobic digestion, archaea are more sensitive to toxic compounds and also exhibit lower growth rates. Acidifiers present ten to twenty-fold higher growth rates and five-fold conversion rates than methanogens [1,69]. With regard to their sensibility toward toxic compounds, methanogens exhibit low tolerance against ammonium nitrogen. Depending on digester pH and operation temperature, the ammonium/ammonia equilibrium might shift. This latter component has been claimed to be highly toxic for methanogens. Ammonia diffuses freely through the permeable membrane of methanogens cells causing changes in intracellular pH and resulting in potassium deficiency and/or proton imbalance [78]. Moreover, ammonium can also inhibit enzymes that are involved in methane production [79]. Yenigün and Demirel [80] reported inhibition of the methanogenesis stage at total ammonia nitrogen (TAN) and ammonia concentrations of 1700–1800 mg/L and 150 mg/L, respectively. As a result, the high concentration of TAN (NH_3_ and NH_4_^+^) can lead to volatile fatty acids accumulation. This last process involves acidification of the anaerobic broth, which in turns inhibits methanogens activity. Therefore, the main drawback of protein rich biomass, such as microalgae, during digestion is the high amount of nitrogen released in form of ammonium that can inhibit methane formation. In fact, this inhibition has been already evidenced by Mahdy et al. [38] during the digestion of protein rich *Chlorella vulgaris*. Those authors attributed the stepwise methane production decrease to the high nitrogen mineralization (77%) taking place during the digestion of protease pretreated microalgae biomass. In this manner, microalgae proteins are not only limiting the hydrolysis stage of the anaerobic digestion but they might also be detrimental in methanogenesis stage. Similar to the developed strategies to overcome the negative effect of microalgae proteins in hydrolysis, some solutions have been proposed to overcome the issues that proteins might cause in methanogenesis during those last years of research.

To avoid inhibition by ammonium, different strategies can be implemented. One of them entails the use of nitrogen poor media for microalgae cultivation. Due to the low nitrogen availability in the medium, proteins accumulation is restricted while lipids and carbohydrates fractions become more abundant in the grown biomass [81,82]. Biogas production was modified using this method in different studies [80,83]. This strategy can be easily applied by using urban wastewater as culture media, which normally contains considerable lower nitrogen concentrations than synthetic salt media (≈60 vs. 300–600 mg N/L). The benefit of this strategy has been evidenced recently using *Spirulina* biomass for biogas production [12]. Similar results were obtained with *C. vulgaris*, where a higher accumulation of carbohydrates (40%) was observed when microalgae was grown in urban wastewater while only 22% was obtained in biomass grown in synthetic medium. Concomitantly with the increase in carbohydrates, protein biomass content was reduced (from 64 to 33%) and thus, methane production was enhanced [40]. 

A second approach to avoid ammonium inhibition is through sludge bioaugmentation. This approach consists in introducing or enriching specific anaerobic microorganisms with special features. Thus, anaerobic microorganisms that are tolerant to high NH_4_^+^ concentrations should be used within the anaerobic sludge to accomplish this goal. Although it is generally believed that total ammonia levels above 3 g/L have toxic effect on the methanogens, the resistance of methanogens can be increased by exposing the microorganisms to high nitrogen concentrations [83]. The use ammonia tolerant inocula has been recently demonstrated as an efficient option for digestion of *C. vulgaris* and cattle manure [84]. In this study, the effectiveness of adapted methanogens resulted in a 33% methane yield increase. This approach allowed operating the digester at 3.7–4.2 g NH_4_^+^-N/L. Tian et al. [85] operated an acclimation experiment in continuous anaerobic reactors fed with substrate rich in the protein fraction such as microalgae and cattle slurry manure. Results showed a stable biomethanization process despite of the high ammonium concentration (10 g NH_4_^+^-N/L). Authors stressed the changes on the anaerobic population taking place as the responsible feature to handle high ammonium concentration. Even though this biological strategy is very promising, it is necessary to do further research due to the challenges that might arise such as the different behavior that the bioaugmented inocula under different operational conditions imposed in the reactors. Attention must be directed to microorganism’s population since they might fail to thrive or be washed out from the reactors.

## 5. Conclusions

Anaerobic digestion of microalgae has been presented as a promising alternative for generation of bioenergy. The implementation of this process requires pretreatment of the rigid algae cell wall in order to make available the organic matter to anaerobes. Enzymatic pretreatment with proteases showed the best performance in terms of organic matter solubilization and methane production. This feature already highlighted the importance of proteins in the hydrolysis stage of anaerobic digestion. Solving this problem with protease addition could result in methanogens inhibition mediated by high ammonium concentrations reached during nitrogen mineralization. Two solutions are proposed to overcome potential inhibition, namely the reduction of nitrogen levels of microalgae biomass using a low nitrogen concentration culture media and the use of ammonium tolerant anaerobic inocula. This fact showed that protein embedded in microalgae cell wall might be responsible for their inherent low biodegradability. Microalgae proteins might be crucial not only in the hydrolytic phase but also during methanogenesis.

## Figures and Tables

**Table 1:** Studied pretreatments to improve biogas production using microalgae as substrates.

**High Demanding Energy Pretreatments**	**Operation Mode**	**Biomass**	**Conditions**	**Methane Yield Increase**	**References**
Thermal	Batch	*Scenedesmus* sp.	75 °C for 10 h 95 °C for 10 h	58% 69%	[29,30,31]
	Batch	*Scenedesmus* sp.	80 °C for 15 min	60%	[32]
	Batch	*Chlorella* sp.	70 °C for 30 min 90 °C for 30 min	37%48%	[33]
	Batch	*Stigeoclonium* sp. *Monoraphidium* sp and *Nitzschia*	130 °C for 15–30 min	28%	[31]
	Semi-continuous	*Chlorella* sp.	120 °C40 min	1.5-fold	[34]
Mechanical	Batch	*Scenedesmus* sp.	128.9 KJ/g TS for 30 min	87%	[32]
	Batch	*Monoraphidium* sp. and *Stigeoclonium* sp.	26.7 KJ/g TS for 30 min	85%	[31]
	Batch	*Mixture of microalgae biomass*	10; 27; 40; 57 KJ/g TS	6-24%	[35]
Chemical	Batch	*Chlorella* sp. and *Scenedesmus* sp.	CaO (4 and 10% w/w) at 25, 55 and 72 °C	25%	[36]
	Batch	*Chlorella* sp.	4 M H_2_SO_4_ at 120 °C for 20–40 min	72.5%	[37]
**Low Demanding Energy Pretreatments**		**Biomass**	**Solubilization**	**Methane Yield**	**References**
Proteases	Batch	*C. reinhardtii* *C. vulgaris*	86-96% for both biomasses	51% in *Chlorella* biomass 7% C. *reindhartii*	[38]
	Batch	*Scenedesmus* sp.	30%	1.53-fold	[39]
	Semi-continuous	*C.vulgaris*	47%	2.6-fold	[39]
	Semi-continuous	*C. vulgaris*	54%	5 and 6.3-fold (OLR= 1.5 g/L d and OLR= 3 g/L d )	[40]
Carbohydrases	Batch	*C. vulgaris* and *Scenedesmus* sp.	84% 36%	1.2-fold	[41]

**Table 2:** Aminoacid products based on Stickland reaction (modified from [73]).

Amino Acid	Formula	HAc	HProp	HBu	HVa	IN	IC	Other	H_2_	ATP
Arginine	C_6_H_14_O_2_N_4_	0.5	0.5	0	0.5	4	1	0	−1	1
Histidine	C_6_H_9_O_2_N_3_	1	0	0.5	0	3	1	1	0	2
Lysine	C_6_H_14_O_2_N_2_	1	0	1	0	2	0	0	0	1
Tyrosine	C_9_H_11_O_3_N	1	0	0	0	1	1	0.882	1	1
Tryptophan	C_11_H_12_O_3_N	0	0	0	0	1	1	1.471	2	1
Phenylalanine	C_9_H_11_O_2_N	0	0	0	0	1	1	1.176	2	1
Cysteine	C_3_H_6_O_2_NS	1	0	0	0	1	1	0	1	1
Methionine	C_5_H_11_O_2_NS	0	1	0	0	1	1	0	1	1
Threonine	C_4_H_9_O_3_N	1	0	0.5	0	1	0	0	−1	1
Serine	C_3_H_7_O_3_N	1	0	0	0	1	1	0	1	1
Leucine/Isoleucine	C_6_H_13_O_2_N	0	0	0	1	1	1	0	2	1
Valine	C_5_H_11_O_2_N	0	0	1	0	1	1	0	2	1
Glutamine	C_5_H_9_O_4_N	1	0	0.5	0	1	1	0	0	2
Aspartate	C_4_H_7_O_4_N	1	0	0	0	1	2	0	2	2
Glycine	C_2_H_5_O_2_N	1	0	0	0	1	0	0	−1	0
Alanine	C_3_H_7_O_2_N	1	0	0	0	1	1	0	2	1
Proline	C_5_H_9_O_2_N	0.5	0.5	0	0.5	1	0	0	−1	0
